# Influence of Sandblasting Process on Tribological Properties of Titanium Grade 4 in Artificial Saliva for Dentistry Applications

**DOI:** 10.3390/ma14247536

**Published:** 2021-12-08

**Authors:** Patrycja Osak, Joanna Maszybrocka, Maciej Zubko, Jan Rak, Sylwia Bogunia, Bożena Łosiewicz

**Affiliations:** 1 Faculty of Science and Technology, Institute of Materials Engineering, University of Silesia in Katowice, 75 Pułku Piechoty 1A, 41-500 Chorzów, Poland; joanna.maszybrocka@us.edu.pl (J.M.); maciej.zubko@us.edu.pl (M.Z.); jan.rak@us.edu.pl (J.R.); 2 Old Machar Medical Practice, 526-528 King Street, Aberdeen AB24 5RS, UK; sylwia.bogunia@nhs.scot

**Keywords:** compressive residual stress, sandblasting, titanium, tribological wear, wettability

## Abstract

Titanium Grade 4 (Ti G4) is widely used in medicine for dental implants. The failure-free life of implants depends on their properties such as resistance to wear and friction processes. This paper presents an analysis of the influence of sandblasting on tribological wear of commercial dental implants made of TiG4 in artificial saliva. Tribological wear measurements were performed in a reciprocating motion in the ball-on-disc system. The scanning electron microscopy/energy-dispersive X-ray spectroscopy (SEM/EDS) method was used to characterize the surface of the implants before and after the tribological wear test. The microhardness of Ti G4 was measured before and after sandblasting by the Vickers method. The contact angle was determined by the method of sitting drop in air. The residual stress test using the X-Ray Diffraction (XRD) single-{hkl} sin^2^ψ method was carried out. The compressive residual stress of 324(7) MPa and surface hardening of Ti G4 was revealed after sandblasting with Al_2_O_3_ particles of 53–75 μm in diameter. It was found that sandblasting changes the surface wettability of Ti G4. The intermediate wettability of the mechanically polished surface and the hydrophobicity of the sandblasted surface was revealed. Sandblasting reduces the tribological wear and friction coefficient of Ti G4 surface in saliva. The three-body abrasion wear mechanism was proposed to explain the tribological wear of Ti G4 in saliva.

## 1. Introduction

Dental implant prosthetics is one of the most dynamically developing fields of modern medicine. The increase in social welfare, the aging society, and a very large increase in dental caries results in an increasing demand for dental implants. Oral diseases affect the quality of life as well as the proper physiology of the oral cavity. Lack of teeth influences chewing disorders, speech problems, and aesthetics [1,2,3]. The solution to these problems is dental implants that supplement single teeth or provide support for permanent dentures–bridges and removable dentures. Dental implants are used when the number of teeth is insufficient for the proper functioning of the oral cavity. They not only maintain the aesthetic effect by imitating normal teeth but also prevent bone loss and avoid changes in the temporomandibular joints. The most important problem limiting the proper functioning of dental implants is wear resistance. Additionally, dental implants must be resistant to friction due to the method of application [4].

Titanium is most often used for dental implants [5,6,7]. It is characterized by high corrosion resistance and good mechanical properties similar to bone tissue. Ti is biocompatible in the human body and does not cause toxic reactions. Considering that one of the factors determining the success of the osseointegration process is the appropriate surface of dental implants, the titanium surface was modified over the years to ensure the appropriate roughness and porosity [6,8,9,10,11]. Originally, machine-surface titanium implants were used with a roughness *Ra* of 0.7–0.8 μm only [9,12,13,14,15]. The surface obtained by machining is characterized by parallel grooves and burrs formed during cutting. The resulting smooth surface extends the initial stabilization time of dental implants [16]. Therefore, a rougher surface is required to stabilize the titanium implants and create a collagen scaffold [15]. The most known is sandblasted surface, which serves both to clean the surface after cutting and to increase the surface roughness. The authors showed a 34% increase in sandblasted surface roughness compared to the machine surface [17]. The sandblasting process consists in bombarding the surface with abrasive particles to obtain a roughness of 1–3 μm. After the squeezing process, the surfaces of dental implants can be additionally etched obtaining the sandblasted and etched surface (Sandblasted Large-grit Acid-eatched, SLA) [18,19], or the double acid-etched surface (DE) [18]. Bioactive coatings on titanium are also currently developed to obtain innovative dental implants [18,20]. The sandblasting can improve the adhesion of such coatings to the substrate and improve the tribological properties and corrosion performance of titanium.

Abrasive wear is one of the most common causes of damage to biomaterials [4,21,22,23]. It increases material consumption and shortens the life of the implants. Therefore, the tribological characteristics of dental materials are crucial in the appropriate selection of materials for the implant, as well as in the appropriate modification of the surfaces of these materials. Degradation of dental implants is related to wear and corrosion factors. Cyclical loads, micromovement of implants in bone tissue, as well as natural biofilm in the oral cavity affect the processes of implant destruction. Tribological wear reduces the corrosion resistance of biomaterials. The abrasive wear is a natural process that affects tooth tissues and crowns mounted on implants. It is caused by surface-to-surface contact, which is closely related to the intensity of the forces applied to the teeth in the mouth to the bone supporting the teeth [24,25]. The tribological and corrosion resistance of dental implants is influenced not only by the surface of the material but also by the biological environment of the oral cavity. Saliva consists of approximately 98% water and 2% inorganic and organic substances and electrolytes. It also contains immunoglobulins and proteins such as mucin, albumin, and lysozyme [21,22,23]. From the point of view of tribological wear, the task of saliva is to reduce friction in the tooth-food and tooth-tooth systems [26,27].

The behavior of the biomaterial in the biological environment depends on the properties of its outer layer, the most important of which are roughness, chemical composition, and wettability [4,6,9,10,12,13,14,24,25,28,29]. The surface roughness of the dental implants influences the interaction between the biomaterial and the tissues affecting the rate of osseointegration [12,13,14,25,28,29]. A macro-rough surface is characterized by a surface development with *Ra* amounting to approximately from a few millimeters to several tenths of a micrometer [12,13,14]. It is a surface with too much development, allowing only mechanical anchoring of the implant, showing difficulties with full osseointegration, as large surface irregularities impede cell growth. The micro-rough surface is characterized by the *Ra* value from 1 to 10 μm, however, the most optimal value is in the range of 1–3 μm [12,13,14,15,18,25]. Such a surface ensures the stability of the implants, supports the osseointegration process, and reduces the risk of ion release with tribological wear. Nano-rough surfaces are defined as surfaces with the *Ra* of less than 1 μm. The surface with such a low roughness makes it difficult to heal the implant, and additionally, as a result of its tribological wear, metal ions are released into the body [6]. The machined smooth surface of the implants is the nano-rough surface [12]. The simplest and most effective method to increase the surface roughness of titanium is sandblasting [18,19,20,25]. In the sandblasting process, the titanium surface is strongly plastically deformed, which causes deformation hardening in the surface layer of sandblasted titanium. Titanium with a sandblasted surface shows about 10% higher fatigue strength at high cycles than titanium with the machined surface. The sandblasting process also increases the corrosion resistance of titanium and thus reduces the tribological wear of this metal [25,30].

Tribological phenomena at the interface between the bone tissue and the implant play an important role. Wear and friction result from the properties of the implant-bone system. Tribological research is necessary to understand the mechanisms of implant degradation in the oral cavity environment, as well as to evaluate newly developed methods of surface modification of dental implants [31]. Titanium shows poor wear resistance [31,32,33]. However, literature reports mainly describe clinical complications of the titanium dental implants used [30]. Close attention is paid to aesthetic complications, but mechanical complications of the use of titanium implants are hardly mentioned by scientific reports. Most of the published works analyze the tribological wear of dental implants without considering a fluid-rich oral environment. Whereas saliva has lubricating properties which are of great importance to reduce friction and wear of implants. To fully analyze the tribological wear, it is necessary to analyze the tribological system consisting of the tested dental implant, a counter-sample simulating a natural tooth, and a liquid that acts as a slip. For dental implants, only use artificial saliva as a lubricant [34]. A load is generated during the tribological wear test. The presence of saliva causes the breakage of the TiO_2_ layer, leading to the degradation of dental implants. As a result of degradation, particles of the implanted material are released, leading to further degradation [35]. The wear mechanism determined during the test has a key role in the prediction of the behavior of biomaterials during functioning in the human body. Therefore, when performing tribological research on implants, it is necessary to recreate the conditions in the human body as much as possible.

The main aim of this study is to determine for the first time the influence of the sandblasting process on the tribological wear of dental implants made of titanium Grade 4 (Ti G4) in artificial saliva. The microstructure, stresses, wettability, and microhardness of Ti G4 before and after the sandblasting process was also the subject of the study.

## 2. Materials and Methods

### 2.1. Preparation of the Material Surface

The subject of the study was Ti G4 in the form of a rod (Bibus Metals, Dąbrowa, Poland) and commercial dental implants produced in the system HEX (Osteoplant Research and Development, Poznań, Poland). The chemical composition and the physical properties of Ti G4 according to ISO 5832–2 [36] and ASTM F67 [37] were given in our earlier publication [24,38,39]. Ti G4 samples were prepared as discs with a diameter of 10 mm and a height of 5 mm. One-sided mirror-like surface of the samples was obtained by grounding with 80 to 2500# grit SiC paper and polishing using final OP-S suspension (Struers, Cleveland, OH, USA). Next, two-step ultrasonic cleaning for 20 min in acetone, and then in ultra-pure water of resistivity 18.2 MΩ cm at 25 °C (Millipore SAS, Molsheim, France) was conducted. The cleaning procedure was carried out twice. Commercial machine-surface implants were only subjected to cleaning.

**Figure 1:**
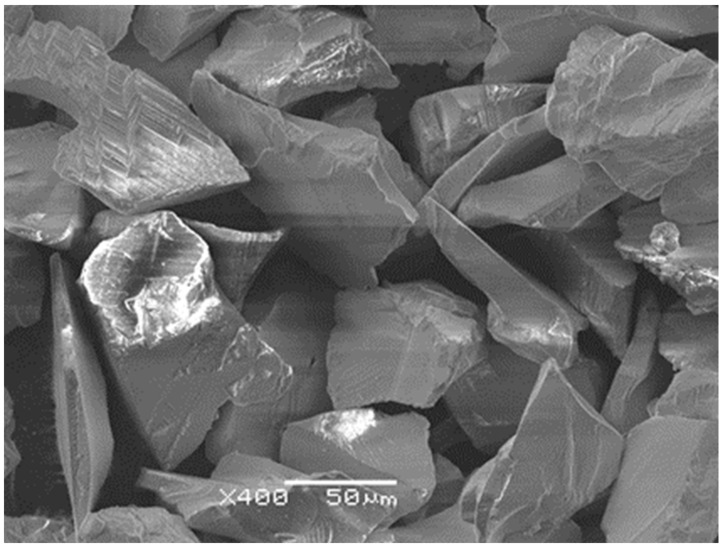
SEM image of surface morphology of Al_2_O_3_ abrasive.

So prepared samples and implants with the machined surface were sandblasted using white Al_2_O_3_ of FEPA Grit F220 [40]. The surface morphology of Al_2_O_3_ grains was examined by scanning electron microscopy (SEM) using a JEOL JSM-6480 microscope (SEM, Peabody, MA, USA) at the voltage of 20 kV and the current intensity of 75 mA (Figure 1). The sharp and polyhedral grains of Al_2_O_3_ are visible, which break according to defined planes during sandblasting and reduce the abrasive wear. The Al_2_O_3_ grain size was in the range of 53 to 75 μm.

The chemical composition of the corundum used was given previously [24]. Sandblasting was carried out using a dental sandblaster AX-B5 4 (Osakadent, Foshan, Guangdong, China) at a pressure of 0.6 MPa, time of 15 s, distance of 1.5 cm between the sandblasting nozzle and the sandblasted surface. Sandblasted Ti G4 samples and implants were sonicated for 20 min in acetone and finally in ultra-pure water to remove Al_2_O_3_ residues from their surface.

The arithmetic mean deviation of the roughness profile of *Ra* = 0.12(1) μm and *Ra* = 1.65(7) μm characterized the mechanically polished and sandblasted surface of Ti G4, respectively [25].

### 2.2. Material Characterization

After the sandblasting process, the residual stresses in the tested material were measured. The X-ray measurements in Bragg–Brentano geometry were carried out using an Empyrean diffractometer (Malvern Panalytical, Malvern, UK) with a Cu anode (CuKα wavelength = 1.54056 Å) with a nickel filter operating at an electric current of 30 mA and a voltage of 40 kV and equipped with a PIXcell^3D^ detector.

The microhardness of the samples was measured before and after sandblasting by the Vickers method with a hardness scale of HV = 0.1 using a Wilson^®^–WolpertTM Microindentation Tester 401MVD (Wilson Instruments, LLC, Carthage, TX, USA). The measurement consisted of pressing an indenter in the form of a regular, quadrilateral diamond pyramid with a dihedral angle α = 136° into the surface of the test sample under the load *F* perpendicular to this surface. After the load was removed, the diagonal was read from the resulting square-shaped imprint. The measurements were performed according to the ISO 6507–1 standard [41]. The requirements for hardness testers and standards are described in ISO 6507–2 [42] and ISO 6507–3 [43], respectively.

Measurements of the contact angle (*Θ*_C_) by the method of sitting drop in air were carried out for Ti G4 samples before and after sandblasting. An OCA 35 goniometer (DataPhysics Instruments GmbH, Filderstadt, Germany) at 21 °C was used. Five drops of ultra-pure water of a volume of about 2 μL each were placed in five places on the surface of the sample. Based on the obtained images, the mean values of the *Θ*_C_ were calculated.

### 2.3. Tribological Characteristics

**Figure 2:**
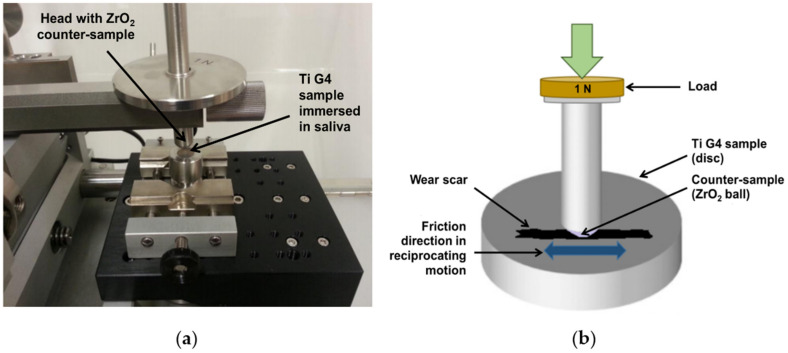
Schematic illustration: (**a**) experimental setup for tribological wear measurements in a reciprocating motion in ball-on-flat system; (**b**) ball-on-disc tribological test principle.

Tribological wear measurements of Ti G4 samples before and after sandblasting were performed in a reciprocating motion in the ball-on-disc system using a tribometer (Anton Paar Poland, Warszawa, Poland), shown in Figure 2a. The ball-on-disc tribological test principle is presented in Figure 2b. As a counter-sample, a ZrO_2_ ball with a diameter of 3 mm was used. The tests were carried out in an artificial saliva solution (ASS) with the chemical composition given in the previous work [24]. The normal force in the friction node was 3 N, the sliding speed was 2.5 cm s^−1^, and the stroke length was 4 mm. The test consisted of 10,000 cycles (back and forth = 1 cycle), corresponding to a total friction distance of 81 m.

The wear analysis of the samples after tribological tests was performed based on the profilometric analysis of the wear scars. A SURFTEST SJ-500 profilometer (Mitutoyo, Sakado, Japan) was used to record the profiles of wear scars. Specific wear rate (*V*_v_) in mm^3^ N^−1^ m^−1^ was determined according to Equation (1) [44]:

(1) $$V_{v}\;=\;\frac{A\cdot l}{F_{n}\cdot s}$$

where: *A* denotes the area of the wear scar in mm^2^, *l* is the stroke length, *F*_n_ is the normal force, and *s* relates to the friction path.

The wear analysis of the ZrO_2_ ball was performed based on measuring the diameter of the wear scar that was formed on the ball by means of a BX51 optical microscope (Olympus, Shinjuku, Tokio, Japan).

## 3. Results and Discussion

### 3.1. SEM Observations before and after Sandblasting

**Figure 3:**
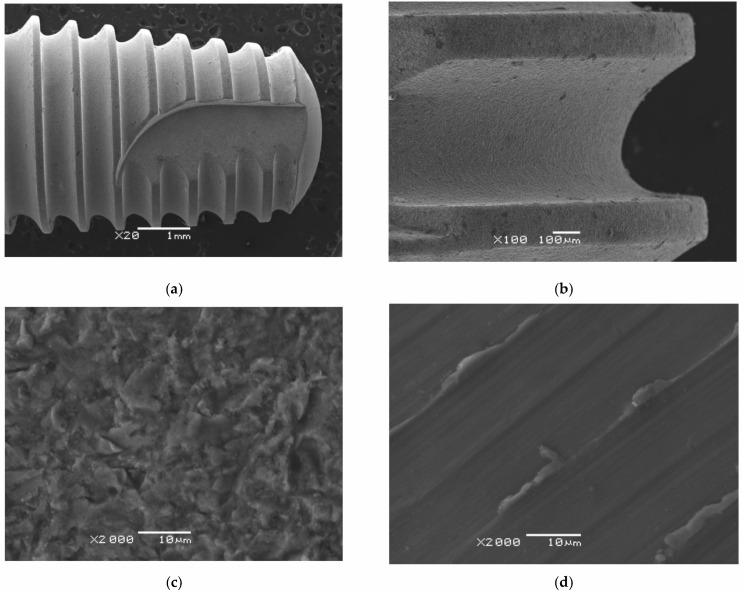
SEM image of a titanium dental implant: (**a**–**c**) sandblasted surface; (**d**) machined surface.

SEM images of the surface morphology of a titanium dental implant with a sandblasted surface at a magnification of 20, 100, and 2000 times are shown in Figure 3a–c. For comparison, the surface morphology of a machined surface at a magnification of 2000 times is presented Figure 3d.

One can see that the machined surface of the Ti G4 implant is poorly developed with visible parallel grooves produced by the manufacturing process of turning, polishing and/or milling (Figure 3d). Remains of molten metal and burrs are visible in the grooves formed after the implant surface treatment. The titanium implant surface is threaded, which increases the metal-to-bone contact surface (Figure 3a,b). The thread improves stability, however, direct bone contact with the machine surface can, over time, create fine gaps between the tissue and the implant. The resulting gaps can lower the mechanical fixation of the implant, and its loss in a later stage. Such a phenomenon is associated with the resistance of osteoblast growth on the resulting surface furrows; in particular, in places of low-quality bone tissue [28,45,46].

The sandblasted surface of the titanium implant is characterized by increased roughness (Figure 3c). On the surface, there is no molten metal residue and no burrs that were removed during sandblasting. On the other hand, there are numerous dents caused by bombardment with corundum particles. There were also microcrackers and microcracks resulting from the residual stresses generated during sandblasting. Such a developed surface topography can directly improve the initial stability of the dental implant and its long-term stability by mechanically anchoring the resulting irregularities in the bone tissue. Research also shows that sandblasted surfaces show much better cell proliferation and differentiation [28,47,48,49]. It was reported in our previous paper that the stresses generated harden the Ti G4 surface and impede the stick of Al_2_O_3_ particles into the surface. We observed this effect for sharp-edged Al_2_O_3_ particles with the optimum grain size of 53–75 μm [25].

### 3.2. Stress and Microhardness Analysis

**Figure 4:**
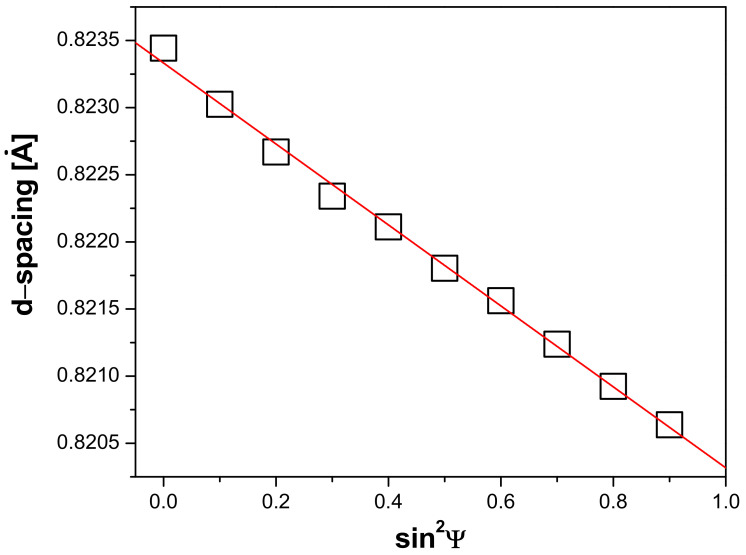
Graph of compressive stresses present in Ti G4 after sandblasting.

Stress in the material is one of the characteristics of the surface properties of the material influencing the success of the implantation process. Therefore, after the sandblasting process, the Ti G4 samples were subjected to residual stress tests using the X-ray diffraction (XRD) single-{hkl} sin^2^ψ method. The (213)-peak of α-Ti at the position of 2*θ*_CuKα_ = 139.5° was chosen for the stress measurements. The constant step of 0.1 in the sin^2^ψ space was used in the 0–0.9 range resulting in the ψ tilt angles of 0°, 18.43°, 26.57°, 33.21°, 39.23°, 45.00°, 50.77°, 56.79°, 63.43°, and 71.57°. The data analysis was performed assuming isotropic elastic constants for α-Ti taken from [50]. The profile of the residual stresses in the sandblasted surface layer on Ti G4 is shown in Figure 4.

The residual stress state in the sandblasted material’s surface was measured to be compressive of 324(7) MPa. The sandblasting process generates a thin layer of compressive residual stresses on the titanium surface. Residual stresses are limited only to a shallow surface layer of approximately 226(7) μm. Stresses are compressive in nature due to plastic deformation caused by the ejection of abrasive Al_2_O_3_ particles under pressure. For comparison, in case of a commercially pure titanium sandblasted with SiO_2_ particles of 200–300 μm in diamater, the compressive residual stress measured by XRD was around 480 MPa [51]. Sandblasting increases the roughness of the surface, and compressive residual stresses cause the increase in adhesion of osteoblasts, as well as more durable and faster osseointegration [52,53]. However, sand bombardment of the surface generates local plastic strains.

**Figure 5:**
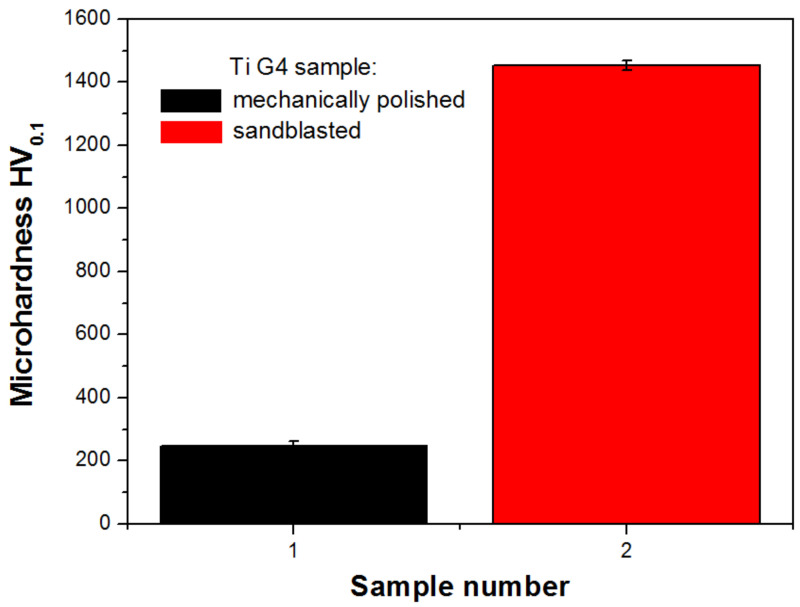
Microhardness of mechanically polished and sandblasted Ti G4.

Both mechanically polished and sandblasted Ti G4 samples were tested for microhardness (Figure 5). The average Vickers microhardness HV_0.1_ is 245.7(17) and 1453(15) for mechanically polished and sandblasted Ti G4, respectively. The almost six-fold increase in the microhardness value after sandblasting is related to the presence of stresses. The hardness of sandblasted titanium in combination with the presence of compressive stresses significantly reduces the fatigue consumption of the material compared to that of the machined surface [54]. In the case of titanium dental implants that are exposed to cyclical loads, an increase in the value of these parameters is desirable. The obtained results are in accordance with data reported for commercially pure titanium sandblasted with SiO_2_ particles in which case the microhardness value of 240 HV for the near surface layer with a depth of 10 μm was determined [51].

### 3.3. Effect of Sandblasting on Surface Wettability

**Figure 6:**
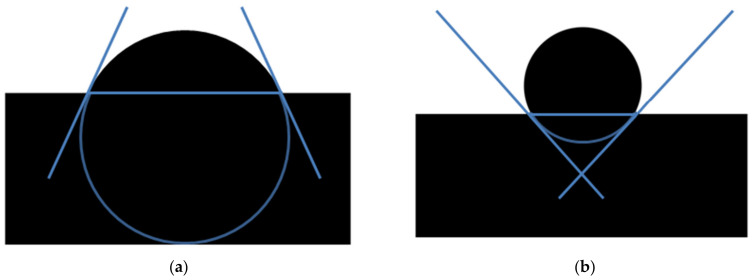
Image of angle formed tangential to water droplet at air–liquid–solid interface for Ti G4: (**a**) mechanically polished; (**b**) sandblasted.

When a solid is wetted by a liquid such as blood, the liquid adheres to the surface of the solid. Figure 6 shows representative images of the angle formed tangential to the water droplet at the air-liquid-solid interface for Ti G4 mechanically polished and sandblasted.

The obtained *Θ*_C_ for Ti G4 after mechanical polishing is 66(2)°, and after sandblasting, it takes the greater value equal to 131(2)°. The obtained results indicate that the sandblasting process, in addition to changes in the surface topography (Figure 3), changes the surface wettability of Ti G4 from intermediate wettability to hydrophobic. However, the sandblasting process is necessary to increase the surface development of dental implants. The contact angle is an important element for accelerating tissue proliferation and the development of bone tissue, especially osteoblasts. Immediately after the implantation procedure, the contact of body fluids, and in particular blood, with the surface of the implant causes repair processes in the body to start through local inflammation and the release of growth factors. The next stage is the migration of proteins and cell adhesion, and finally the formation of osteoblasts [55].

### 3.4. Tribological Characteristics

The tests of resistance to tribological wear and measurements of the friction coefficient were carried out under conditions of sliding friction in the presence of artificial saliva. Ti G4 was subjected to tribological tests after the mechanical polishing and sandblasting. A summary of the parameters obtained during the ball-on-disc tribological test is presented in Table 1. Before and after the wear test, an analysis of the wear of the counter-sample, i.e., the wear scar of the ZrO_2_ ball, was performed. Based on microscopic observations, the values of *d*_1_ and *d*_2_ diameters, and the average value of the diameter of the *d*_av_ were determined. The *d*_av_ for mechanically polished TiG4 is 630 μm and it decreases to 441 μm after sandblasting. Based on the average value of the ball wear scar, the specific wear of the ball, *V*_b_ was determined, which for the polished Ti G4 is 2.17·10^−5^ mm^3^ N^−1^ m^−1^ and it significantly decreases to the value of 5.19·10^−6^ mm^3^ N^−1^ m^−1^ in the case of the sandblasted Ti G4.

**Figure 7:**
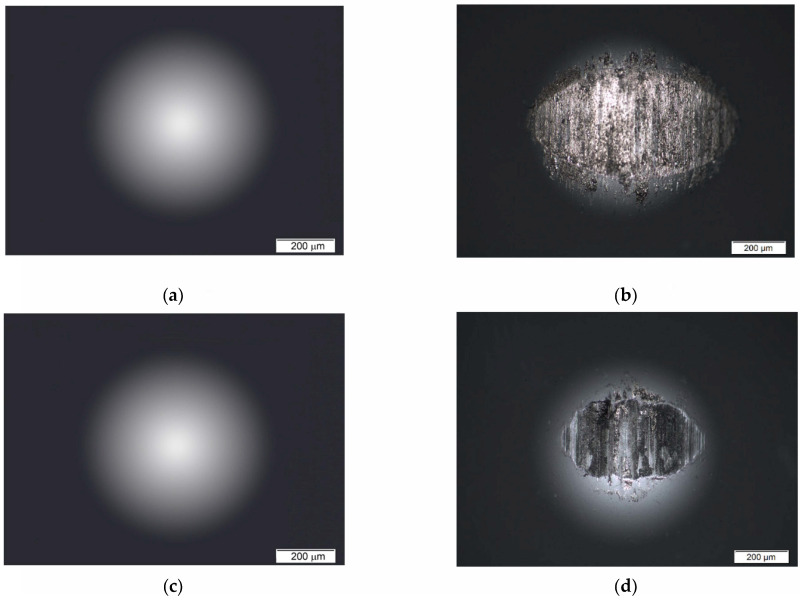
Wear scar of ZrO_2_ ball after ball-on-disc tribological test against Ti G4: (**a**) after mechanical polishing before test; (**b**) after mechanical polishing after test; (**c**) after sandblasting before test; (**d**) after sandblasting after test.

The microscopic images of the ZrO_2_ ball wear scar before and after ball-on-disc tribological test against mechanically polished and sandblasted Ti G4 are presented in Figure 7. The direction of damage on the surface of the balls is visible from top to bottom (Figure 7b,d). After the tribological test for mechanically polished Ti G4, residual abrasion of the material is visible on the surface of the ball (Figure 7b). The ball wear scar for sandblasted Ti G4 is characterized by the lack of residual traces of the tested material, which proves lower tribological wear of titanium after sandblasting (Figure 7d).

The sandblasting process reduces wear due to friction of cooperating elements. Sandblasted titanium surface with the addition of a biological lubricant in the form of artificial saliva reduces the friction parameters. The wear scar for mechanically polished Ti G4 is characterized by numerous tears on the ball surface (Figure 7b), while after sandblasting the surface of the wear scar is smoother (Figure 7d). During the wear test, the titanium particles were abraded and constituted an additional surface destructive factor, resulting in an increase in friction due to surface polishing. In the case of sandblasted Ti G4, in the first stage, the irregularities formed on the surface, i.e., the so-called peaks, were rubbed off. In the next step, the surface was polished by worn particles. The wear scar of the sandblasted surface is much narrower and shallower, with less impurities transferred to the surface of the ZrO_2_ ball, compared to the wear scar of the mechanically polished surface (Figure 7).

**Figure 8:**
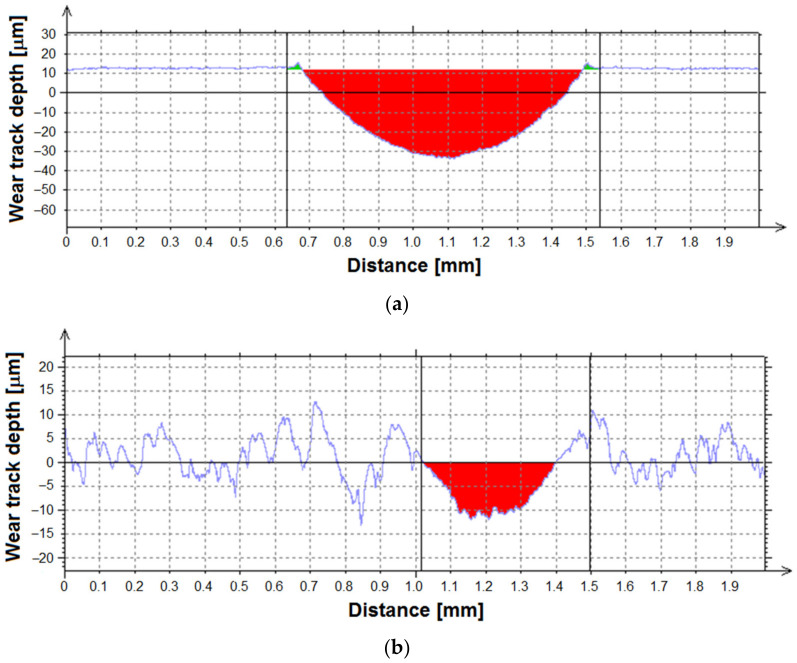
Cross-sectional profiles of wear tracks after ball-on-disc tribological test for Ti G4: (**a**) after mechanical polishing; (**b**) after sandblasting.

The surface morphology strongly influences the tribological wear of titanium. The sandblasted Ti G4 surface shows less material wear in comparison with the machined surface, as evidenced by the obtained cross-sectional profiles of wear tracks after the ball-on-disc tribological test (Figure 8).

The diameters of wear scars in Figure 7 are corresponding to the width of wear tracks after the ball-on-disc tribological test shown in Figure 8. The width of the wear track for Ti G4 after mechanical polishing is 838(6) μm and only 294(3) μm after sandblasting. The average wear surface area (*A*_av_) after the tribological test is 24,923 μm^2^ for mechanically polished Ti G4 and is more than 8 times higher compared to *A*_av_ for sandblasted Ti G4 (Table 1). The average material volume consumption (*V*_m_) calculated based on *A*_av_ was 4.15·10^−4^ mm^3^ N^−1^ m^−1^ for Ti G4 after mechanical polishing and 5.01·10^−5^ mm^3^ N^−^^1^ m^−^^1^ for Ti G4 subjected to sandblasting (Table 1). The obtained results show that the sandblasting process significantly reduces the wear of titanium surfaces of dental implants in the presence of saliva.

**Figure 9:**
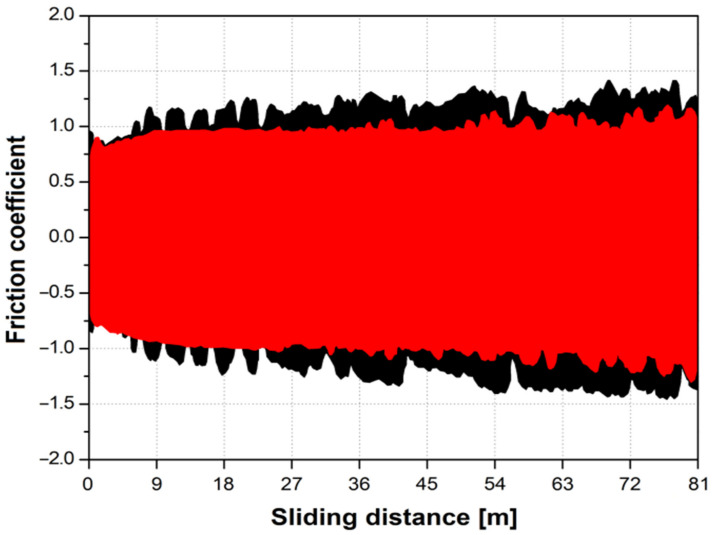
Static friction coefficient as a function of sliding distance for Ti G4 mechanically polished (black) and sandblasted (red).

Figure 9 shows the course of the static friction coefficient as a function of the sliding distance for mechanically polished (black) and sandblasted (red) Ti G4.

**Figure 10:**
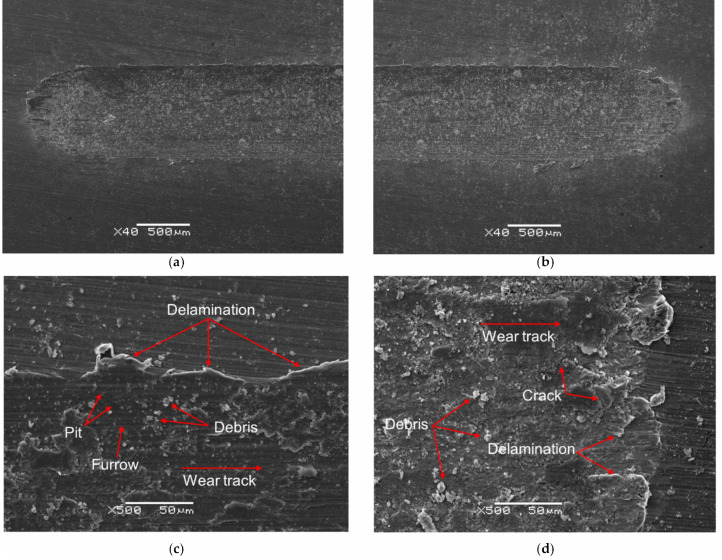

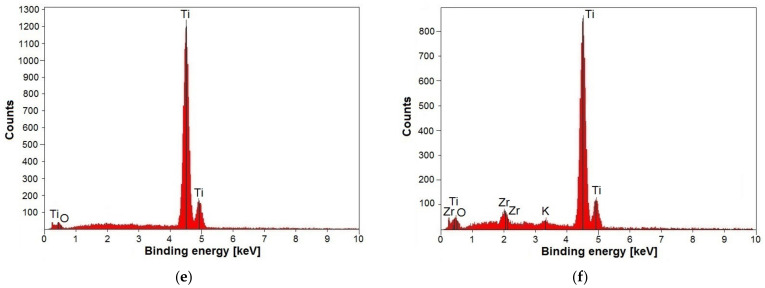
Scanning Electron Microscope (SEM) image of wear tracks for mechanically polished Ti G4 after ball-on-disc tribological test: (**a**,**b**) top-view; (**c**,**d**) border of wear track and surface, and energy-dispersive X-ray spectroscopy (EDS) spectrum from micro-area for: (**e**) untested Ti surface and (**f**) center region of wear track.

**Figure 11:**
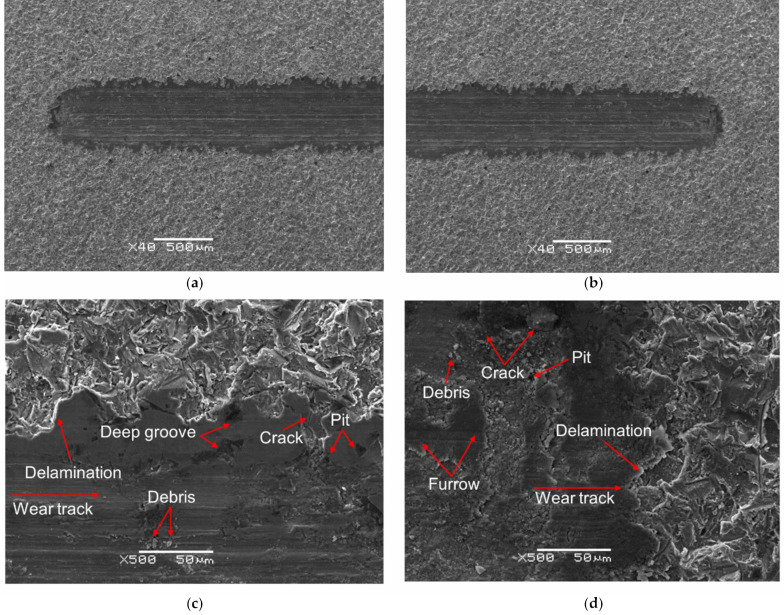

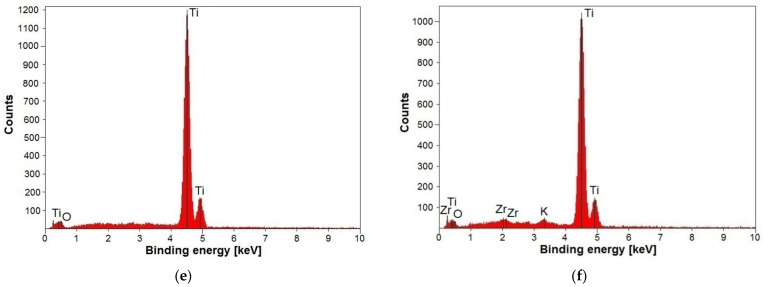
SEM image of wear tracks for sandblasted Ti G4 after ball-on-disc tribological test: (**a**,**b**) top-view; (**c**,**d**) border of wear track and surface, and EDS spectrum from micro-area for: (**e**) untested Ti surface and (**f**) center region of wear track.

The coefficient of friction represents the resistance of the material to friction against the counter-sample penetrating the material. The lower the friction coefficient, the greater the scratch resistance [56]. Based on the course of the friction coefficient, the static friction coefficient, μ_s_ was determined for Ti G4 after mechanical polishing 1.20(28) and after sandblasting 1.03(04) (Table 1). The μ_s_ depends on the applied force. The value of the kinetic friction coefficient, μ_k_ for Ti G4 before and after sandblasting is 0.96(10) and 0.86(06), respectively (Table 1). The μ_k_ is determined from the moving sample. The values of the friction coefficients strongly depend on the type of the tested surface and the test conditions. Lower values of μ_s_ and μ_k_ indicate that in the case of a sandblasted Ti G4 sample, the frictional resistance is reduced, and the wear of the mating elements is limited. Sandblasting surface irregularities create reservoirs in which lubricant is collected in the form of artificial saliva. During the friction process, the biological lubricant is extracted from the cavities of the surface roughness, it is spread over the surface, reducing wear and frictional resistance. The containers created in the sandblasting process allow for the accumulation of wear products (debris) generated during the friction process, so that they do not generate additional wear. The above observations can be applied to the literature on textured surfaces for technical applications [57]. It is generally accepted that the surface texture promotes lubrication by changes in the flow and thickness of the lubricating fluid film, both locally and in the entire contact area. The surface texture act as grease supply channels to contacting surfaces. In Figure 9, for Ti G4 after sandblasting, characteristic areas can be distinguished on the graph of the friction coefficient as a function of sliding distance. In the first test area, up to approx. 40 m of the test, the course of the curve is stable, as evidenced by a smooth curve, followed by ever greater fluctuations in the friction coefficient. Such a course of the curve is the result of wear of the sandblasted layer, which is confirmed by microscopic observations of the wear track (Figure 10 and Figure 11).

The tribological wear characteristics of dental metal biomaterials such as 316L steel, NiCrMo alloy, technically pure titanium (ASTM-grade 2) and Ti6Al4V ELI alloy (ASTM-grade 5) was performed in artificial saliva but with a different chemical composition according to ISO 10,271 and pH of 5.3 [58]. The resistance to wear was determined in the ball-on disc test using a counter sample in the form of Al_2_O_3_ under a load of 5N on the total distance of 100 m. A comparison of the determined friction coefficients demonstrated lower values of mean friction coefficient (higher resistance to wear) in comparison with those for the mechanically polished and sandblasted Ti G4 in the following order: NiCrMo alloy (μ = 0.317) < Ti6Al4V alloy (μ = 0.361) < Ti grade 2 (μ = 0.451) < 316L steel (μ = 0.538). All tested materials exhibited an abrasive wear nature. However, abrasive wear was dominant for Ti grade 2 and Ti6Al4V alloy, while adhesive phenomena connected with plastic deformation of secondary wear products were observed for NiCrMo, Ti6Al4V and Ti grade 2.

SEM images of the wear track after the ball-on-disc tribological test for mechanically polished Ti G4 are presented in Figure 10a–d. Comparative SEM images of the wear track for sandblasted Ti G4 are shown in Figure 11a–d.

SEM analysis of the surface of the wear tracks for the mechanically polished and sandblasted Ti G4 shows that the dominant mechanism is abrasive wear, additionally intensified by micro-cutting visible in the form of continuous scratches in both cases. Scratches and furrows resulting from the movement of loose wear products (debris) along the Ti–ZrO_2_ cooperation track are visible. In addition, single pits also occur. The presence of transverse microcracks indicates the fatigue mechanism for both types of the Ti surface. One can also see oxides giving bright reflections in the SEM image in the microareas where delamination occurred. The surface of titanium was subjected to systematic loads during the tribological wear test. The particles of titanium and the ZrO_2_ ball formed during wear created a kind of load-carrying abrasive. This resulted in the accumulation of plastic strains and stresses on the titanium surface, causing it to crack. During the 10,000-cycle test, loads are generated cyclically, and cracks may grow [59]. As a result of the final loads, the fragments of the oxide film separated to form thin patches at the interface between the sample and the wear track, and separated material particles. The sandblasted surface, as a result of tribological wear, showed more microcracks caused by a microhardness, which was almost six-times higher (Figure 5). During the wear test, first, roughness peaks formed in the sandblasting process were sheared, and only later did the matrix plastic deformation take place. After the process of lapping the friction surfaces, the contact area between the cooperating surfaces increased. The effect of the surface cooperation was the formation of cracks and deep groves filled with the remains of cooperating elements and a solution of artificial saliva acting as a biological lubricant. A smaller amount of debris on the narrower wear track is observed for the sandblasted Ti G4 surface, which points to the reduction of tribological wear as a result of sandblasting.

The EDS spectrum from the micro-area for the untested Ti G4 surface after mechanical polishing and center region of the wear track is presented in Figure 10e,f, respectively. The corresponding EDS spectra for untested Ti G4 surface after sandblasting are presented in Figure 11e,f. The obtained EDS spectra for both types of Ti G surface show differences in terms of quality and quantity. On the EDS spectrum shown in Figure 10e and Figure 11e, the presence of peaks originating only from the Ti G4 sample is confirmed. The surface contents of elements determined based on the peaks identified is 66.7 at.% for Ti, and 33.3 at.% for O in Figure 10e, and 45.2 at.% for Ti, and 54.8 at.% for O in Figure 11e. In case of the EDS spectra from the micro-area at the center region of the wear track in Figure 10f and Figure 11f, the additional peaks from Zr and K are revealed. Based on the peaks identified, the following surface contents of elements was determined Ti: 41.6(8) at.%, O: 56.0(9) at.%, Zr: 1.5(3) at.%, K: 0.9(1) at.% in Figure 10f, and Ti: 31.9 at.%, O: 63.5 at.%, Zr: 2.5 at.%, K: 2.1 at.% in Figure 11f. Zr-peaks correspond to the ZrO_2_ ball wear products while K-peaks come from a solution of artificial saliva. A decrease in atomic concentration for Ti is due to the tribological wear.

EDS method was used as a micro-analytical technique conventionally used SEM for the local determination of chemical elements on self-passivated metal surface [60]. The EDS microanalysis was regarded as a non-destructive technique because the CpTi G4 implant prior to analysis did not differ from the implant after the analysis. The accuracy of calculated concentrations of individual elements was affected by the accuracy of the relative intensity of all the elements in the specimen. In general, the EDS method operates on the hypothetical composition of the sample, i.e., without light elements or depending on the stoichiometry, and equates the results of the spectrum measurements to 100% of the total composition. However, the oxygen content on the surface containing the TiO_2_ oxide layer on the surface of the tested implants had to be considered in the real analysis of the EDS results. The EDS quantitative research method cannot be considered as routine like other spectroscopy-chemical methods.

### 3.5. Wear Mechanism of Ti G4 in Artificial Saliva

**Figure 12:**
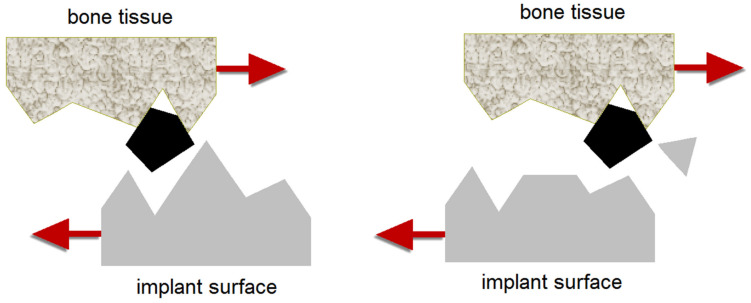
Three-body abrasion wear mechanism of dental implant surface from titanium debris.

The obtained characteristics of tribological wear for titanium implant in artificial saliva prove the wear mechanism of three-body abrasive wear (Figure 12). In this mechanism, the wear particles act as an abrasive between the surface of the bone tissue and the surface of the implant. Wear refers to the progressive loss of implant material from interacting surfaces when the parts are in motion against each other. The three-body abrasion wear mechanism has more practical significance than the two-body abrasion wear mechanism but receives less attention [61,62,63]. The rate of material wear in three-body abrasion is one order of magnitude lower in comparison with that for two-body abrasion. This is because in the three-body abrasion wear mechanism abrasive grains can move freely, which does not always cause wear. Abrasive grains can roll and tumble along the implant surface, rather than sliding around and cutting furrows and deep grooves. They can also align presenting the bluntest profile to the surface.

In the case of mechanically polished Ti, the smooth surface of the material contributes to high tangential friction, while sandblasting unevenness makes the tangent sections of the sample with the counter-sample much smaller despite the same force pressure in the friction node. In three-body abrasion wear mechanism of titanium implant, the particles of the abraded Ti are free to roll and slide on the surface. Sandblasting generates stress strains inside the material, which results in low abrasion. In the case of dental implants, faster tribological wear can lead to a reduction in the quality of bone tissue or its loss, and thus the loosening of the implant. Consequently, the dental implant has to be removed. Tribological wear of dental implants can be reduced by surface treatment in the sandblasting process [14,24,51]. The smooth surface wears out more quickly, also leading to corrosion of the materials. Sandblasting is a simple but effective way to reduce tribological wear of the surface of dental implants and at the same time increases the properties of corrosion resistance in a biological environment. Saliva as a biological lubricant in the tooth biotribological system plays an extremely important role [24,26,31,38,64]. The presence of saliva reduces both the friction coefficient and the wear rate of the titanium implant. Such a behavior is due to the decrease in the adhesion of the two surfaces of bone tissue and implant surface in contact as well as the interaction between the two asperities.

## 4. Conclusions

Based on the obtained results, the sandblasting process with Al_2_O_3_ particles of 53–75 μm in diameter is an effective method of modifying the surface of Ti G4 used for dental implants by increasing its surface roughness, microhardness, and resistance to tribological wear in saliva. Sandblasting is one of the fastest and most economical ways to produce a rough surface, however, it affects the change in the wettability of the Ti G4 surface. The intermediate wettability of the mechanically polished surface and the hydrophobicity of the sandblasted surface were demonstrated. The compressive residual stress caused by sandblasting was determined by the X-Ray Diffraction (XRD) single-{hkl} sin^2^ψ method to be about 324(7) MPa.

Based on the obtained tribological characteristics of mechanically polished and sandblasted Ti G4 in saliva in a reciprocating motion in the ball-on-flat system, sandblasting reduces the wear of the elements cooperating in the friction process and reduces the coefficient of static and kinetic friction. Based on microscopic analysis of wear scar of ZrO_2_ ball after the ball-on-disc tribological test against Ti G4 and cross-sectional profiles of wear tracks, the sandblasted Ti G4 surface showed less material wear in comparison with that of the mechanically polished surface. The *d*_av_ decreases from 630 to 441 μm and the width of the wear track from 838 to 294 μm after sandblasting. The sandblasting process strongly influences the decrease of the specific wear of the ball from 2.17·10^−5^ mm^3^ N^−1^ m^−1^ for the mechanically polished Ti G4 to 5.19·10^−6^ mm^3^ N^−1^ m^−1^ for the sandblasted Ti G4. As a result of sandblasting, the average material volume consumption calculated based on *A*_av_ also decreases from 4.15·10^−4^ mm^3^ N^−1^ m^−1^ for Ti G4 after mechanical polishing to 5.01·10^−5^ mm^3^ N^−1^ m^−1^ for Ti G4 subjected to sandblasting. The reservoirs formed as a result of bombardment with Al_2_O_3_ particles create cavities on the titanium surface, in which lubricant in the form of saliva accumulates. During friction, this biological lubricant is released from the reservoirs, reducing the wear of the titanium surface. In addition, the surface roughness of Ti G4 causes abrasion of the surface vertices firstly, and then of the deeper parts of the material. The three-body abrasion wear mechanism may be proposed as the main mechanism to explain the tribological wear of the titanium implant in saliva.

## Figures and Tables

**Table 1:** Parameters obtained after ball-on-disc tribological test for Ti G4 after mechanical polishing and sandblasting.

Ti G4	*d*_av_ [μm]	*V*_b_ [mm^3^ N^−1^ m^−1^]	*A*_av_ [μm^2^]	*V*_m_ [mm^3^ N^−1^ m^−1^]	μ_s_	μ_k_
Mechanically polished	630	2.17·10^−5^	24,923	4.15·10^−4^	1.20(28)	0.96(10)
Sandblasted	441	5.19·10^−6^	3003	5.01·10^−5^	1.03(04)	0.86(06)

## Data Availability

Not applicable.
